# Domain Analysis and Motif Matcher (DAMM): A Program to Predict Selectivity Determinants in *Monosiga brevicollis* PDZ Domains Using Human PDZ Data

**DOI:** 10.3390/molecules26196034

**Published:** 2021-10-05

**Authors:** Haley A. Wofford, Josh Myers-Dean, Brandon A. Vogel, Kevin Alexander Estrada Alamo, Frederick A. Longshore-Neate, Filip Jagodzinski, Jeanine F. Amacher

**Affiliations:** 1 Department of Chemistry, Western Washington University, Bellingham, WA 98225, USA; haley.wofford@gmail.com (H.A.W.); vogelb2@wwu.edu (B.A.V.); estradk@wwu.edu (K.A.E.A.); longshf@wwu.edu (F.A.L.-N.); 2 Department of Computer Science, Western Washington University, Bellingham, WA 98225, USA; myersdj@wwu.edu (J.M.-D.); jagodzf@wwu.edu (F.J.)

**Keywords:** protein–protein interactions, PDZ domains, choanoflagellates, evolution, target selectivity, protein–peptide interactions, signaling

## Abstract

Choanoflagellates are single-celled eukaryotes with complex signaling pathways. They are considered the closest non-metazoan ancestors to mammals and other metazoans and form multicellular-like states called *rosettes*. The choanoflagellate *Monosiga brevicollis* contains over 150 PDZ domains, an important peptide-binding domain in all three domains of life (Archaea, Bacteria, and Eukarya). Therefore, an understanding of PDZ domain signaling pathways in choanoflagellates may provide insight into the origins of multicellularity. PDZ domains recognize the C-terminus of target proteins and regulate signaling and trafficking pathways, as well as cellular adhesion. Here, we developed a computational software suite, Domain Analysis and Motif Matcher (DAMM), that analyzes peptide-binding cleft sequence identity as compared with human PDZ domains and that can be used in combination with literature searches of known human PDZ-interacting sequences to predict target specificity in choanoflagellate PDZ domains. We used this program, protein biochemistry, fluorescence polarization, and structural analyses to characterize the specificity of A9UPE9_MONBE, a *M. brevicollis* PDZ domain-containing protein with no homology to any metazoan protein, finding that its PDZ domain is most similar to those of the DLG family. We then identified two endogenous sequences that bind A9UPE9 PDZ with <100 μM affinity, a value commonly considered the threshold for cellular PDZ–peptide interactions. Taken together, this approach can be used to predict cellular targets of previously uncharacterized PDZ domains in choanoflagellates and other organisms. Our data contribute to investigations into choanoflagellate signaling and how it informs metazoan evolution.

## 1. Introduction

PDZ domains are small peptide-binding domains named for the proteins where they were first discovered: PSD-95, Dlg1,and ZO-1 (PDZ) [1,2,3,4]. These are scaffolding domains that recognize the extreme C-terminus of target proteins, with the bulk of the PDZ–protein interaction involving up to 6 residues on the target [5,6,7]. PDZ domains are found in all three domains of life (Archaea, Bacteria, and Eukarya), and PDZ-mediated interactions are important in signaling and trafficking pathways, as well as in cell adhesion [5,8,9]. Examples include: regulation of the trafficking of receptors throughout the cell, e.g., the cystic fibrosis transmembrane conductance regulator (CFTR), signaling pathways mediated by G-protein coupled receptors (GPCRs), and interactions with proteins involved in tight junctions and within the postsynaptic densities of neurons [10,11,12,13,14,15,16,17,18,19,20,21,22,23,24,25,26,27]. Due to their central role in cellular processes, PDZ domains are therapeutic targets in a number of human diseases [28,29,30,31,32]. They are also a common target of viral proteins during infection. The best studied examples include PDZ domain targeting by oncoproteins in human papillomaviruses, but several other viruses, including influenza, human immunodeficiency virus (HIV), and coronaviruses also target PDZ domains [5,33,34,35,36,37,38]. For example, proteins in the SARS-CoV-2 proteome, which causes COVID-19 disease, interact with and bind several PDZ domains during infection [39,40,41,42]. The trafficking of angiotensin-converting enzyme 2 (ACE2) receptor, which is targeted by SARS-CoV-2, contains a PDZ-binding motif at its C-terminus (sequence: DVQTSF, motif residues are underlined) and is also regulated by PDZ domains [43].

**Figure 1:**
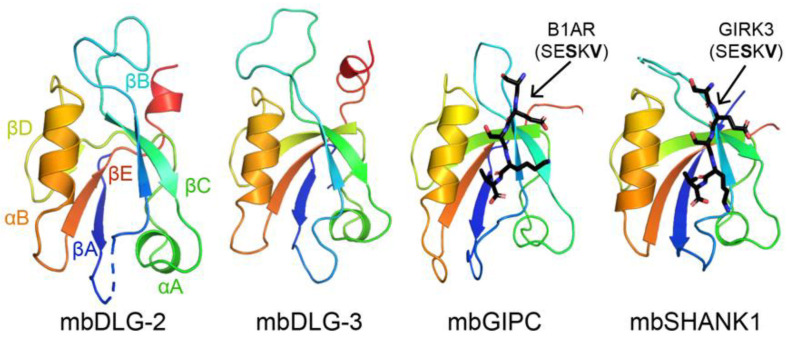
Structures of PDZ domains from *Monosiga brevicollis* are shown in cartoon representation, with the conserved secondary structure elements labeled in the mbDLG-2 figure. Bound peptides are in black sticks and colored by heteroatom (N = blue, O = red) and labeled. PDB ID codes for choanoflagellate PDZ domains from this work include: 6X1X, 6X20, 6X22, 6X23, 6X1P, 6X1N, and 6X1R [48].

There are approximately 270 PDZ domains in the human proteome, and PDZ domain-containing proteins have 1–13 PDZ domains [5]. All PDZ domains characterized to date share a common structural fold, consisting of an antiparallel β-sheet (βA-βE) and 1–2 α-helices (αA–αB) (Figure 1) [5]. Historically, PDZ domains are separated into binding classes, dependent on two positions: the most C-terminal residue, termed P^0^, and two adjacent, or P^−2^ [5,44]. For example, Class I PDZ domains recognize the motif X-S/T-X-Φ, where X = any amino acid and Φ = hydrophobic amino acid (typically, F/I/L/V) [5,6,7,44]. However, these motifs are unable to describe the overlapping specificities of the PDZ family [5]. Over the past decade, we and others have teased apart selectivity determinants at positions other than those described in the characterized motifs [5,45,46,47]. The ability to identify and determine selectivity determinants in closely-related PDZ domains is critical to understanding their role in cellular pathways and processes.

Choanoflagellates are microbial eukaryotes that live in marine and freshwater environments [49]. They are considered the closest relative to metazoans, and evolutionary biologists argue that these organisms can provide insight into the origins of multicellularity [49,50]. For example, choanoflagellates have extensive signaling networks and form a molecular architecture that resembles a multicellular state, called rosettes [51,52,53]. In our previous work, we concluded that there are 178 PDZ domains in *M. brevicollis*, with 1–20 PDZ domains in a single protein [48]. We previously used protein biochemistry and structural biology to characterize several PDZ domains from the *M. brevicollis* proteome, including those from mbDLG, mbGIPC, and mbSHANK1 (Figure 1) [48,54]. These PDZ domain-containing proteins all have clear homologues in the human proteome, and we found that selectivity determinants are largely conserved, despite ~750 million years of evolution between these organisms [48].

In this work, we wanted to expand the investigation of choanoflagellate PDZ domains and develop techniques to characterize those that do not have homologues in the human proteome. Based on our previous work and the work of others, we hypothesized that binding preferences in PDZ domains are directly determined by the amino acids that interact with specific peptide positions at both motif and modulator (or non-motif) positions [45,46,47,55,56,57,58]. Because we aimed to identify endogenous interactions, we chose not to use common high-throughput techniques to determine PDZ selectivity, e.g., phage display or peptide arrays, which are tuned to look for highest affinity binders [32,38,47,59,60]. We reasoned that this approach may limit the number of matching sequences in the *M. brevicollis* proteome and cause us to overlook sequences with lower affinity. It is well established that endogenous PDZ interactions are not necessarily optimized for affinity, as these are often regulatory in nature and in pathways involving several proteins and molecular states [5]. Therefore, in our approach, we developed a computational program, Domain Analysis and Motif Matcher (DAMM), which determines the human PDZ domain(s) that share the greatest number of conserved residues in the peptide-binding cleft with an input PDZ sequence, independent of overall sequence identity. The target sequences of human PDZ domains are largely known; therefore, we then predicted potential target sequences in the choanoflagellate proteome using the Motif Matcher part of the DAMM, based on our published MotifAnalyzer-PDZ program [54]. We used protein biochemistry, structural biology, and DAMM to identify two *M. brevicollis* sequences that bind to a previously uncharacterized PDZ domain with <100 μM affinity, validating our approach. We argue that this computational and experimental pipeline provides a straightforward methodology for predicting cellular targets of PDZ domains and can be applied to other peptide-binding domains, e.g., SH2 and SH3 domains, that also contain conserved structural folds.

## 2. Results

### 2.1. Identification of Peptide-Interacting Residues in PDZ Domains

We wanted to develop a computational pipeline that could be used in combination with knowledge of human PDZ domain specificity to predict the endogenous targets of a PDZ domain that does not have a homologue in *Homo sapiens*. Based on our previous work characterizing PDZ domains in the *Monosiga brevicollis* proteome, we chose to focus on UniProt ID A9UPE9_MONBE, a 545 amino acid protein with one PDZ and one SH3 domain. We will refer to this protein as A9UPE9 moving forward for simplicity. Notably, there are no proteins in the human proteome that contain this domain architecture, and a BlastP search of the full-length sequence returns alignments limited to one of the isolated domains.

**Figure 2:**
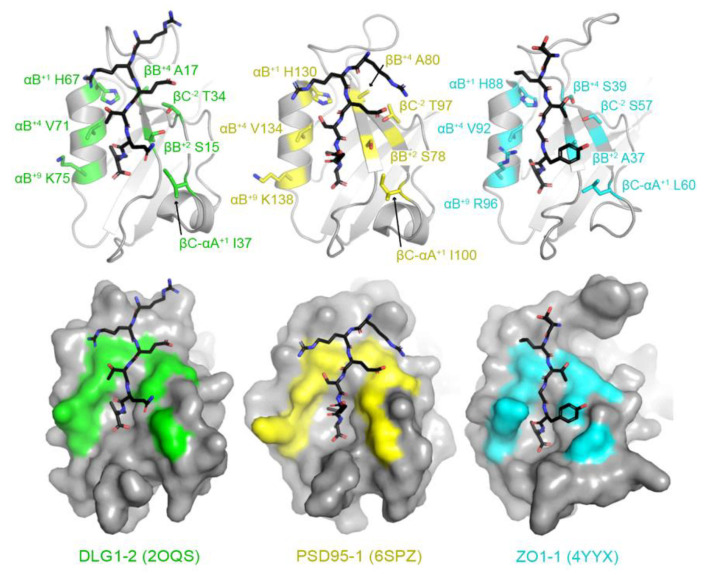
Identification of peptide-binding cleft residues in DLG1 PDZ2 (or DLG1-2, PDB ID 2OQS, green), PSD95-1 (6SPZ, yellow), and ZO1-1 (4YYX, cyan). Proteins are shown in both cartoon (top) and surface representations (bottom) with bound peptides in black sticks and colored by heteroatom (N = blue, O = red). The peptide-binding cleft residues used in this analysis are represented as side chain sticks and are colored by heteroatom or monochrome, respectively, and labeled. DLG1-2 is bound to a peptide of the HPV18 E6 oncoprotein (sequence: RRETQV) [61]. PSD95-1 is bound to a peptide with sequence RRESEI [62]. ZO1-1 is bound to the Claudin2 C-terminal tail (SLTGYV) [63].

Then, using structural analyses of peptide-bound human PDZ domains, we identified seven key residues whose side chains interact directly with the peptide. Examples include peptide-bound structures of DLG1 PDZ2 (or DLG1-2), PSD95-1 (which is also referred to as DLG4-1), and ZO1-1 (Figure 2) [61,62,63]. These include: the βB^+2^, βB^+4^, βC^−2^, βC-αA^+1^, αB^+1^, αB^+5^, and αB^+9^ residues, where the secondary structure elements are as defined in Figure 1, the superscript numbers indicate residues from the N- (positive values) or C-termini (negative values) of those secondary structure elements, and “βC–αA” is the loop between the βC strand and αA helix. Notably, due to a high degree of variability in the length of the βB–βC loop amongst PDZ domains, we did not include residues in this structural element, although they can interact with peptide positions, e.g., P^−4^ and P^−5^. In addition, other residues make non-covalent interactions with peptide residues in individual PDZ domains, but we kept our assignments relatively conservative, limited to those that form the core of the peptide-binding cleft (Figure 2).

**Figure 3:**
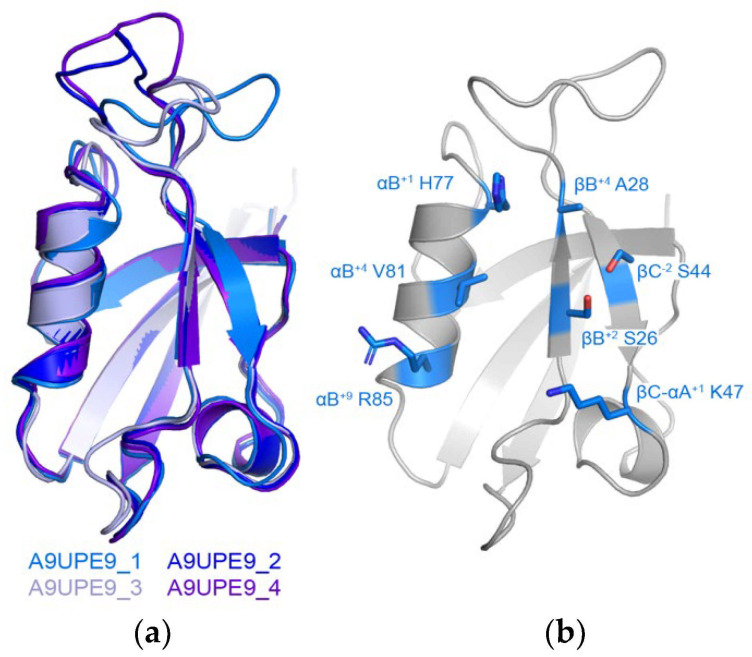
Model of A9UPE9 PDZ structure. The four conformations from Robetta are in cartoon representation and colored as labeled in (**a**). (**b**) Identification of peptide-binding cleft residues in A9UPE9 conformation 1 (A9UPE9_1). Residues used for analysis are shown as side chain sticks, colored marine by heteroatom (N = blue, O = red) and labeled.

We also wanted to use the online Robetta server for de novo structure prediction to generate a model of A9UPE9 (Figure 3) [64]. Robetta output four conformations that mainly differed in the conformation of the βB–βC loop, and alignments revealed RMSD values for main chain atoms, as compared with conformation 1 of 0.455 Å for 289 atoms (conformation 2), 0.530 Å for 291 atoms (conformation 3), and 0.679 Å for 311 atoms (conformation 4) (Figure 3a). Due to the similarities, we restricted our structural analyses to conformation 1. The structure, as well as sequence patterns, allowed us to identify the relevant peptide-binding cleft residues (Figure 3b).

### 2.2. Domain Analysis and Motif Matcher (DAMM) Program

**Figure 4:**
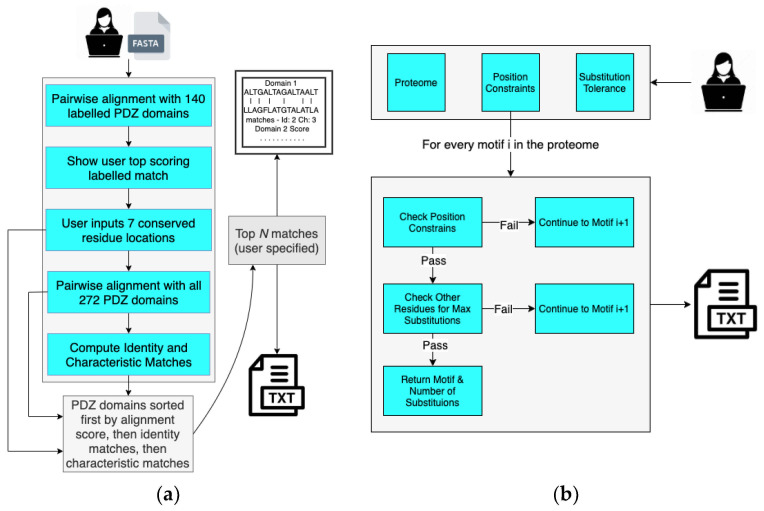
The Domain Analysis and Motif Matcher (DAMM) software suite. Details of the computational workflow of the Domain Analysis program are in (**a**) and Motif Matcher program are in (**b**).

In order to determine the human PDZ domain with the most similar peptide-binding cleft as A9UPE9, we developed a Python-based software suite called Domain Analysis and Motif Matcher (DAMM). The computational pipeline of DAMM is in Figure 4a. We will refer to the input sequence as “A9UPE9” or “the choanoflagellate PDZ domain sequence”, but this program can theoretically be used for any PDZ domain sequence from any organism. Ultimately, we did not want to require a structural model of an input PDZ domain sequence in order to identify the seven peptide-binding cleft residues. Therefore, we manually identified these amino acids for 140 solved PDZ domain structures, based on our previous curation of mammalian PDZ domains in the Protein Data Bank [5]. These data became an input database for our computational program, and the first step in DAMM is to take a single input PDZ domain sequence (<100 residues) in FASTA file format and run a BLAST alignment with the 140 PDB sequences that we manually curated [5,65]. The top pairwise alignment based on sequence similarity is provided as an output with asterisks marking the peptide-binding cleft residues of the annotated PDB structure. The user PDZ domain input sequence has reference numbers, allowing the user to identify and input the seven peptide-binding cleft positions for matching. For example, for A9UPE9, if the input sequence included the N-terminus of the protein, the user would input: 26, 28, 44, 47, 77, 81, and 85 (Figure 3b). It is important to note that these numbers may vary depending on the input sequence, e.g., if the input FASTA file only contained the UniProt annotated A9UPE9 PDZ domain (residues 12–99), the numbers would be shifted by 12 as a result because E12 would be considered residue 1 by DAMM. This description is to highlight that the identified peptide-binding cleft residues will likely not correspond to the residue numbers in the full-length protein.

Following identification of the peptide-binding cleft residues for matching, DAMM runs BLAST pairwise sequence alignments with all previously curated 272 human PDZ domain sequences, including those that do not have a solved structure in the Protein Data Bank [5]. The user is prompted to specify how many sequences to output, listed in descending order by overall sequence identity. Included in the output file is a list of the pairwise alignments with number of peptide-binding cleft matches, in both identity and similarity, indicated for each of the alignments. The output file is ordered by sequence identity to ensure that alignments listed at the top are those where the majority of the PDZ domain sequences aligned, but overall, sequence identity does not play a role in number of matches specified. Amino acids are defined as “similar” based on the following categories, using one-letter abbreviations: acidic (D, E), basic (H, K, R), polar (C, M, N, Q, S, T), non-polar (A, I, L, V), and aromatic (F, W, Y).

Once these results are obtained, the user can run literature and database searches to identity potential binding sequences based on known human PDZ targets. The Motif Matcher piece of DAMM and *MotifMatcher-PDZ* can then be used similarly, where C-terminal sequences in the choanoflagellate proteome are filtered and screened for closest matches to an input sequence [54]. Alternatively, we can use DAMM to screen the *M. brevicollis* proteome for specific preferences at each position, similar to those that would be identified using phage display or another high-throughput technique. All components of DAMM were developed in Python 3, and are available at https://pdzselectivity.cs.wwu.edu (accessed on 11 September 2021).

### 2.3. Binding Preferences of A9UPE9 PDZ

To identify the binding preferences of A9UPE9 PDZ, we used the DAMM program to find the human PDZ domains with the most similar binding cleft residues. For reference, the PDZ domains with the top 5 highest overall sequence identities are in Table 1.

Based on the overall sequences, A9UPE9 appears to most closely resemble the GRIP PDZ domains (Table 1). However, the results from the DAMM program revealed a different result. Table 2 shows the human PDZ domains with the most similar peptide-binding clefts as A9UPE9 PDZ. For reference, the GRIP1-2 and A9UPE9 PDZ domains have one identical residue amongst the peptide-binding cleft positions, an αB^+1^ His, and three similar residues, at the βC^−2^ (Ser for A9UPE9, Thr for GRIP1-2), βC-αA^+1^ (Lys and Arg, respectively), and αB^+9^ (Arg and Lys, respectively). This result highlights the importance of using number of matches and not sequence identity for this type of analysis.

Our DAMM program results clearly show that while the top result for A9UPE9 PDZ is SYNJ2BP PDZ, overall, its peptide-binding cleft is most similar to the DLG family of PDZ domains. Of these PDZ domains, those from DLG proteins are more extensively studied, including by our lab. Therefore, we chose to use DLG1-1 PDZ in fluorescence polarization experiments to directly compare binding affinity values with A9UPE9 PDZ.

**Figure 5:**
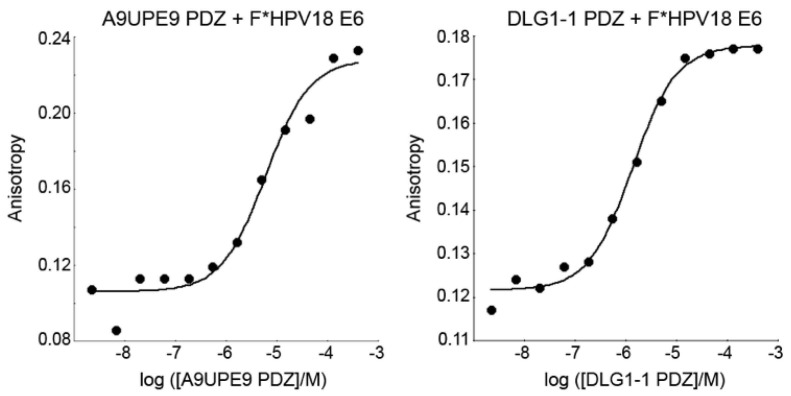
Representative fluorescence polarization experiments to determine *K*_D_ values of A9UPE9 (**left**, as labeled) and DLG1-1 (**right**, as labeled) PDZ domains. Overall *K*_D_ values were calculated from triplicate experiments.

The A9UPE9 and DLG1-1 PDZ domains were expressed and purified following similar protocols, as previously described and in the Materials and Methods [48,54]. The reporter peptide used for both proteins was a fluoresceinated sequence matching the C-terminus of the HPV18 E6 oncoprotein (sequence: FITC-RLQRRRETQV), or F*-HPV18 E6. Determined *K*_D_ values for each protein following triplicate experiments were 2.3 ± 1.6 μM for DLG1-1 PDZ and 6.9 ± 1.7 μM for A9UPE9 PDZ (Figure 5). The HPV18 E6 sequence was chosen based on its known interaction with DLG1-1 PDZ [66,67,68]. A fluoresceinated version of the HPV16 E6 C-terminal sequence (FITC-SSRTRRETQL) was also tested and bound A9UPE9 PDZ with *K*_D_ = 19.5 ± 1.8 μM, based on duplicate experiments. Therefore, we used the higher affinity F*-HPV18 E6 as our reporter peptide.

**Figure 6:**
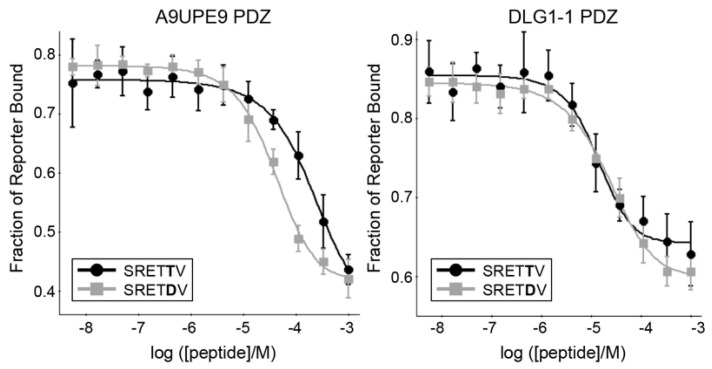
The binding isotherms from competition fluorescence polarization experiments of A9UPE9 (**left**, as labeled), and DLG1-1 (**right**, as labeled) PDZ domains are shown with the HPV18 E6 variant peptides SRETTV and SRETDV. The average values are shown, with error bars representing standard deviation from at least triplicate experiments. *K*_I_ values were determined using SOLVER, as previously described [12,45,46,48,54,60].

We next wanted to interrogate preferences at specific positions along the peptide, as well as to broadly compare binding affinities of known PDZ targets to the A9UPE9 and DLG1-1 PDZ domains. Starting with the HPV18 E6 C-terminal sequence, we designed a number of peptides to test specific peptide positions, including HPV16 E6, HPV18 E6, SRETTV, SRETDV, RRETTV, and RRETDV, where underlined residues differ from HPV18 E6. As a control, we also tested human SNX27, SHANK1, and GIPC PDZ targets previously studied, including GIRK3 (sequence: ESESKV), BPIX (WDETNL), GAIP (QSSSEA), TYRP1 (PNQSVV), and B1AR (ASESKV), as well as previously identified mbSHANK targets, including A9V7Z4 (EDETAL), A9UP44 (QSESRL), and A9UXE1 (QDETAL) [48,54]. Example competition experiment binding curves are in Figure 6, and all *K*_I_ binding affinity values are reported in Table 3.

**Figure 7:**
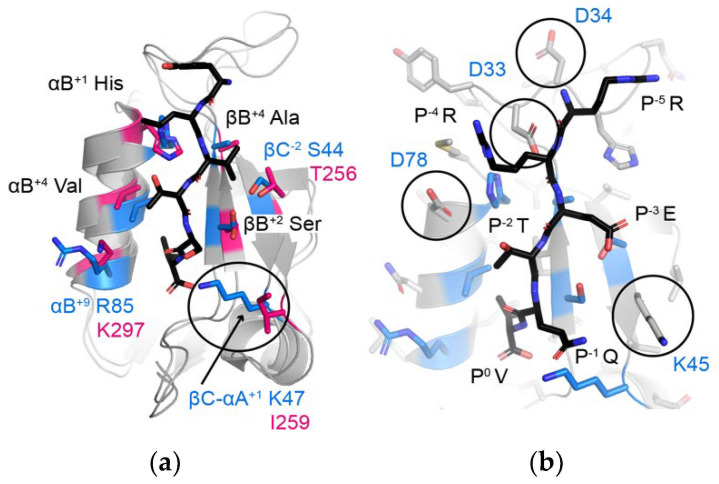
(**a**) Comparison of the peptide-binding clefts of A9UPE9 PDZ (marine) and DLG1-1 (pink, PDB ID 3RL7). DLG1-1 is bound to the peptide matching the C-terminal sequence of the adenomatous polyposis coli (APC) tumor suppressor protein (sequence: YLVTSV) [69]. Peptide-binding cleft residues are shown as side chain sticks, colored by heteroatom (N = blue, O = red) and labeled, where black text indicates shared residues and blue/pink text indicates differences. The βC-αA^+1^ residue, which influences P^−1^ selectivity, is indicated with a black circle. (**b**) Additional residues in A9UPE9 that may influence peptide preferences include D33, D34, K45, and/or D78, which are labeled and highlighted with circles. All side chains are shown as gray sticks and colored by heteroatom, with peptide-binding cleft residues used in the DAMM program colored as in (**a**). The HPV18 E6 peptide (sequence: RRETQV) from PDB ID 2OQS is shown as reference and labeled.

As expected, both A9UPE9 and DLG1-1 bound the HPV18 E6 variant peptides with much higher affinity than peptides matching SNX27, SHANK1, and GIPC targets (Table 3). Our competition experiments revealed a key difference between the A9UPE9 and DLG1-1 PDZ domains. Specifically, while DLG1-1 PDZ bound the HPV18 E6 variant peptides containing a P^−1^ Thr residue with higher affinity, A9UPE9 PDZ greatly preferred a P^−1^ Asp (Table 3). Sequence and structural analyses suggest this difference is due to the βC-αA^+1^ residue, which is I259 in DLG1-1 and K47 in A9UPE9 PDZ and which can directly interact with the P^−1^ residue (Figure 7a). This βC-αA^+1^ Ile is also in DLG1-2 and PSD95-1 (Figure 2). In fact, a multiple sequence alignment of the 12 PDZ domains in DLG1/2/3/4 confirms that these proteins share almost identical peptide-binding clefts, with βB^+2^ = S or N, βB^+4^ = A or V, βC^−2^ = S or T, βC-αA^+1^ = I or L, αB^+1^ = H, αB^+5^ = V or A, and αB^+9^ = K.

Finally, we predicted that the apparent preferences of A9UPE9 PDZ for P^−4^ and P^−5^ Arg residues (e.g., for P^−5^, in RRETDV as compared with SRETDV) are due to D33 and/or D34, residues in the βB–βC loop, as well as D78, the amino acid following the αB^+1^ His, and that a preference for a P^−3^ Glu (as in the HPV sequences) is likely due to K45 (Figure 7b). Taken together, our DAMM program successfully identified DLG PDZ domains as having similar binding preferences as A9UPE9 PDZ, based on shared binding cleft residues. A small number of substitutions in a target sequence, here HPV18 E6, were able to further refine these preferences for A9UPE9 PDZ, and we identified two sequences, F*-HPV18 E6 and RRETDV, that bound with <10 μM affinity.

### 2.4. Endogenous Targets of A9UPE9 PDZ

Finally, we set out to identify potential endogenous targets of A9UPE9 PDZ, based on peptide-binding cleft similarities with human PDZ domains. We used our HPV18 E6 variant sequences (Table 3), as well as known human targets of the top hit from the DAMM program, SYNJ2BP PDZ. As previously described, Motif Matcher scans a filtered set of *M. brevicollis* sequences consisting of the final 6 C-terminal residues for each protein in the UniProt-downloaded proteome for most-similar matches to each sequence [54]. We initially required the program to maintain a P^0^ Val residue, based on the affinity difference between the HPV16 E6 and HPV18 E6 sequences, but allowed P^−2^ to be Ser or Thr. In addition, we ran *M. brevicollis* proteins that were closest matches to known SYNJ2BP PDZ targets, including TMIGD1 (sequence: HSETAL), DLL1/DLL4 (VIATEV), and SYNJ2BP (ASGSSV), through Motif Matcher [70,71,72]. We ultimately tested seven additional peptides in our fluorescence polarization assay, including A9V6G5_MONBE (HRESTV), A9UWH1_MONBE (STRSDV), A9UR52_MONBE (SRRTEV), A9UWP5_MONVE (GSESSV), and A9UYY4_MONBE (RLASEV), as well as A9VB85_MONBE (ARESEI), which contained P^−1^/P^−3^ Glu residues and a P^−4^ Arg, as well as the DLL1/DLL4 sequence directly (Table 4). A9VB85_MONBE was identified by using Motif Matcher to search for sequences with the highest number of positive modulators for A9UPE9 based on our biochemical analyses, including P^−1^ = E/D/Q/N, P^−3^ = E/D, P^−4^ = R/K, and P^−5^ = any residue except E/D.

As shown in Table 4, most of our peptides failed to bind A9UPE9 PDZ with reasonable affinities to suggest an endogenous interaction. Structural analyses suggested that A9UWH1_MONBE and A9UR52_MONBE likely failed to bind due to the presence of the P^−3^ Arg residue, which may negatively interact with the positively-charged K45 (Figure 7b). It is not immediately clear why A9UYY4_MONBE bound with no detectable affinity, defined as >1000 μM. One hypothesis is that A9UPE9 PDZ requires a P^−3^ Glu for binding. Despite containing all other preferred selectivity determinants, including P^−1^/P^−3^ Glu residues and a P^−4^ Arg, the P^0^ Ile in A9VB85_MONBE likely explains why that sequence does not bind A9UPE9 PDZ. Our previous work investigating the basis of P^0^ selectivity found that the PDZ-defining carboxylate-binding loop sequence “GFGF”, as in A9UPE9 PDZ, will likely only accommodate Val and Leu residues [46].

Overall, this work shows that it is challenging to predict endogenous targets of a choanoflagellate PDZ sequence that is not homologous to human PDZ domains. However, we identified one sequence, A9V6G5_MONBE, that binds with an endogenously relevant affinity, <100 μM, using the DAMM program. In addition, a previously tested *M. brevicollis* peptide, A9UP44 (QSESRL), bound A9UPE9 PDZ with *K*_I_ = 64 μM (Table 3). It is not clear why A9UPE9 PDZ bound this sequence with relatively high affinity, despite the P^−1^ Arg residue, and suggests that the P^−3^ Glu provides the majority of free energy of binding amongst non-motif/modulator residues. In our previous work, we successfully used MotifMatcher-PDZ to identify sequences that bound mbSHANK with ~40–70 μM affinity, which is in the same relative range and may reflect the relatively low affinities that PDZ domains have for their endogenous targets [5,54]. Taken together, structural and sequence analyses, in combination with the DAMM program, allowed us to identify two potential endogenous targets of A9UPE9 PDZ. Future work to validate these targets in vivo, as well as to test additional *M. brevicollis* sequences is necessary to develop a holistic view of the cellular function of A9UPE9.

## 3. Discussion

Characterization of the proteins in choanoflagellate signaling pathways can provide important insights into the evolution of human cellular processes. We developed a program to predict the target selectivity of PDZ domains in choanoflagellates, based on conserved amino acids in the peptide-binding cleft. Another critical consideration in future studies will be to think about the other domains in PDZ domain-containing proteins and how they coordinate to regulate cellular processes. For example, there are eight proteins in the *M. brevicollis* proteome that contain both PDZ and SH3 domains, a domain architecture that is only present in choanoflagellates based on BLAST searches, including A9UNP0_MONBE, A9UPE9_MONBE, A9UPI8_MONBE, A9UUC6_MONBE, A9UYE7_MONBE, A9V111_MONBE, A9V6P1_MONBE, and A9V7E4_MONBE. Notably, proteins that contain both PDZ and SH2 domains appear to be unique to organisms from the phylum Choanozoa as well, and there are five *M. brevicollis* proteins that contain these domains: A9V6T4_MONBE, A9V7X5_MONBE, A9VA09_MONBE, A9VB90_MONBE, and A9VC25_MONBE [48].

SH2 and SH3 domains were first discovered in the late 1980s, based on homology between the Src oncoprotein and phospholipase c, and play important scaffolding roles in signal transduction pathways [73,74,75]. There are >120 SH2 domains in the human proteome and >300 SH3 domains [76,77]. SH2 domains recognize phosphotyrosine-containing peptide motifs [78,79,80,81]. SH3 domains recognize proline-rich peptides that form a polyproline type II helix [82]. These domains play critical roles in tyrosine signaling pathways in mammalian cells, by coupling tyrosine phosphorylation to intracellular signaling [83,84,85,86,87].

Work over the past decade has identified a complex network of tyrosine kinase signaling in choanoflagellates, more so than in any metazoan characterized [88,89,90,91,92]. It is clear that such networks are critical for cellular communication, environmental adaptation, and other processes in both non-metazoans, such as choanoflagellates, as well as in metazoans [90]. However, the intricacies of metazoan signaling pathways are not well understood, and future investigations into how PDZ and SH2/SH3 domains act synergistically in these proteins may provide deeper insight into the role of tyrosine kinase signaling in non-metazoans.

**Figure 8:**
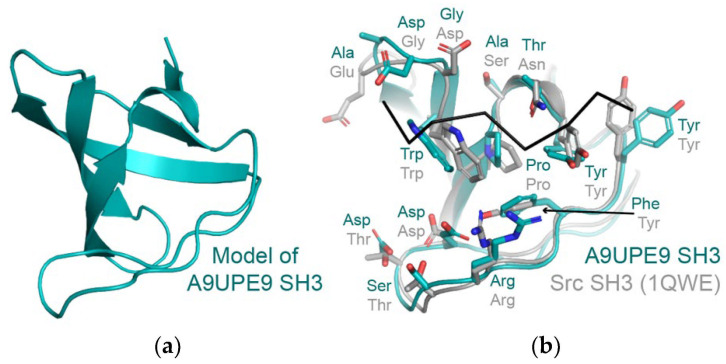
(**a**) A homology model of A9UPE9 SH3 is shown in cartoon representation. The template PDB ID used was 7CSO (Ephexin4 SH3). (**b**) The A9UPE9 and Src SH3 domains are in cartoon representation, with peptide-binding cleft residues in sticks, colored by heteroatom (N = blue, O = red) and labeled. The APP12 peptide is in black ribbon. Alignment of main chain atoms revealed an overall RMSD = 0.824 Å for 170 atoms.

We developed the DAMM program, which can be used to characterize a previously uncharacterized PDZ domain, using sequence identity at key residues in the peptide-binding cleft. We hypothesize that the same approach could be applied to SH3 and other domains, based on conservation in amino acids that directly interact with a peptide target (Figure 8a,b. For example, despite only 29% sequence identity (14/48 residues), structural analyses of the A9UPE9 SH3 and human Src SH3 domains reveal similar peptide-binding cleft residues (Figure 8b). While protein biochemistry can tease apart unique selectivity determinants and identify potential protein–protein interactions in choanoflagellates, cell-based experiments to directly test predictions and investigate these complex pathways will be an exciting next area of research in understanding the evolution of PDZ and tyrosine kinase signaling.

## 4. Materials and Methods

### 4.1. Protein Expression and Purification

Expression and purification of all human and *M. brevicollis* PDZ domains followed a similar protocol, as previously reported for mbSHANK1 PDZ [48,54]. Briefly, N-terminal His-tagged versions of A9UPE9 PDZ (residues 7–98) and DLG1-1 PDZ (residues 220–317) with cleavable TEV sites were inserted into the pET28a+ vector by gene synthesis (GenScript) and expressed in *Escherichia coli* BL21 (DE3) cells. Cells were lysed on ice using sonication. The lysis buffer used was 50 mM Tris pH 8.5, 200 mM NaCl, 10 mM CaCl2, 10 mM MgCl2, 20% (*w*/*v*) glycerol, 50 mM imidazole pH 8.5, 0.25 mM TCEP, DNAse, and protease inhibitor cocktail. Immobilized metal-affinity chromatography, 5 mL HisTrap (GE Healthcare, now Cytiva), was used to purify proteins from the clarified supernatant. The wash buffer used was 25 mM imidazole pH 8.5, 25 mM Tris pH 8.5, 250 mM NaCl, 10% (*v*/*v*) glycerol, and 0.25 mM TCEP, and the elution buffer was 400 mM imidazole pH 8.5, 25 mM Tris pH 8.5, 50 mM NaCl, 10% (*v*/*v*) glycerol, and 0.25 mM TCEP. Except for the protein used in binding experiments, the protein was then dialyzed in dialysis/gel filtration buffer (25 mM Tris pH 8.5, 150 mM NaCl, 10% (*w*/*v*) glycerol, 0.5 mM TCEP) and incubated at 4 °C overnight with TEV protease to cleave off the His-tag. The cleaved protein was then purified using a second nickel column with the wash and elution buffers described above. All proteins were further purified on a Superdex S75 column (Cytiva), using gel filtration buffer. Proteins were concentrated using Amicon centrifugal concentrators (3 MWCO). Concentrated proteins used in fluorescence polarization assays were flash frozen in liquid nitrogen for storage at −80 °C. The extinction coefficient values used for quantification of the proteins at A_280_ were 1490 cm^−1^·M^−1^ (A9UPE9) and 4470 cm^−1^·M^−1^ (DLG1-1).

### 4.2. Fluorescence Polarization

Fluorescence polarization assays were conducted, as previously described [12,45,46,48,54,60]. The reporter peptide used in competition experiments was F*-HPV18 E6 (FITC-RLQRRRETQV), and *K*_D_ values were averaged from triplicate experiments. In both *K*_D_ and *K*_I_ experiments, 30 nM reporter peptide was used. Determined *K*_D_ values for each protein following triplicate experiments were 2.3 ± 1.6 μM for DLG1-1 PDZ and 6.9 ± 1.7 μM for A9UPE9 PDZ. The final protein concentrations used for *K*_I_ competition experiments were 10.5 μM (A9UPE9) and 4.5 μM (DLG1-1). Competition experiments were performed in at least triplicate, and binding affinities were determined using SOLVER, as previously described [12,45,46,48,54,60]. Binding curves were visualized using Kaleidagraph.

### 4.3. Design and Development of Domain Analysis and Motif Matcher (DAMM)

The DAMM software suite was written using Python 3. The first step of DAMM, Domain Analysis (DA), receives a PDZ domain sequence in FASTA format as input from the user. DA interfaces with BioPython and performs a pairwise BLAST-style alignment of the input sequence to the 140 PDZ-labeled domains in our database [93]. DAMM outputs the top-scoring pairwise alignment, showing known conserved residues, then prompts the user for a list of seven conserved residues. Critically, the sequence will not properly align with the annotated PDZ domain sequences if the input is >100 residues, so it is important to input just the PDZ domain itself.

Following identification of peptide-binding cleft residues, DA aligns the input sequence with the sequences of all 272 human PDZ domains, based on previous curation [5]. The output file lists pairwise alignments in order based on BlastP score and also lists the number of matching and/or similar peptide-binding cleft residues. Amino acid similarity is based on the following five groupings: (group 1, hydrophobic) AILV; (group 2, negatively-charged) DE; (group 3, positively-charged) KRH; (group 4, aromatic) FYW; (group 5, polar) CMNQST. Glycine (G) and proline (P) are not included in the groupings because of their unique chemical and/or structural properties.

A user might want to perform a motif similarity search after the alignment analysis and determination of number of matching/similar peptide-binding cleft residues. DAMM provides a separate motif matching program, inspired by *MotifAnalyzer-PDZ* and here named Motif Matcher (MM) [54]. Motif Matcher takes as input a target proteome, specific position constraints for each motif residue (e.g., P^0^ = F, I, L, or V and P^−2^ = S or T), and the maximum amount of substitutions a user will tolerate (e.g., for a hexamer with two defined motif positions, the maximum number here would be equal to four). The output is a list of all C-terminal hexameric sequences that meet these constraints, including the number of substitutions at non-motif positions (Table 5).

### 4.4. Protein Analyses

Structural models of A9UPE9 PDZ were created using the Robetta online server [64]. A structural homology model of A9UPE9 SH3 was created using SwissModel [94,95,96]. Sequence alignments were performed using BLAST and T-coffee [65,97]. All structure figures were created using PyMOL.

## 5. Conclusions

Here we provide an experimental pipeline to predict endogenous targets of a previously uncharacterized PDZ domain with no homology to any human protein. In our approach, we use structural conservation and sequence analyses to determine positional selectivity determinants, and we successfully identify two choanoflagellate sequences with endogenously relevant binding affinities. We argue that this type of approach is applicable to other peptide-binding domains, e.g., SH2 and SH3 domains. These experiments have the potential to provide important insights into signaling pathways in choanoflagellates and other organisms, allowing us to better understand the origins of multicellularity.

## Figures and Tables

**Table 1:** Highest sequence identity values of human PDZ domains with A9UPE9 PDZ.

PDZ Domain	Sequence Identity	Sequence Similarity
INADL-10	42% (35/83)	59% (49/83)
SNTG2	41% (36/87)	57% (50/87)
GRIP1-2	41% (30/74)	60% (45/74)
GRIP2-2	41% (26/63)	61% (39/63)
GRIP1-4	35% (30/85)	51% (44/85)

**Table 2:** DAMM program results for A9UPE9 PDZ.

PDZ Domain	Matching/Similar Residues	Sequence Identity
SYNJ2BP	5/2	31%
DLG1-2	4/2	29%
DLG2-2	4/2	29%
DLG4-2	4/2	29%
MPDZ-12	4/2	32%
DLG3-2	4/2	30%
DLG1-1	4/2	30%
DLG2-1	4/2	29%
DLG4-1	4/2	29%
DLG3-1	4/2	30%

**Table 3:** Binding affinities for the A9UPE9 and DLG1-1 PDZ domains, determined by fluorescence polarization experiments. For all, F*-HPV18 E6 was used as a reporter peptide, and values reflect at least triplicate experiments. Differences as compared with HPV18 E6 are underlined, as relevant. Lines indicate related clusters of peptides: HPV sequences, HPV18 E6 variant sequences, SNX27/SHANK1/GIPC targets, and mbSHANK *M. brevicollis* targets, respectively.

		*K*_I_ Values (μM)	
Peptide	Sequence	A9UPE9 PDZ	DLG1-1 PDZ
HPV18 E6	RLQRRRETQV	16 ± 1	2.8 ± 1.3
HPV16 E6	SSRTRRETQL	66 ± 24	23 ± 5
SRETTV	SRETTV	83 ± 31	4.4 ± 1.9
SRETDV	SRETDV	12 ± 3	7.9 ± 3.7
RRETTV	RRETTV	33 ± 1	2.0 ± 1.6
RRETDV	RRETDV	8.1 ± 3.1	9.5 ± 6.6
GIRK3	ESESKV	>1000	350 ± 20
BPIX	WDETNL	330 ± 160	460 ± 140
GAIP	QSSSEA	>1000	980 ± 180
TYRP1	PNQSVV	97 ± 24	46 ± 21
B1AR	ASESKV	95 ± 30	44 ± 23
A9V7Z4	EDETAL	580 ± 160	460 ± 190
A9UP44	QSESRL	64 ± 35	39 ± 25
A9UXE1	QDETAL	460 ± 210	190 ± 20

**Table 4:** Binding affinities for A9UPE9 PDZ with *M. brevicollis* sequences, determined by fluorescence polarization experiments. The reference sequence for Motif Matcher is included, where applicable. Experiments conducted as in Table 3 and as described in the Materials and Methods.

			*K*_I_ (μM)
Peptide	Sequence	Reference Sequence	A9UPE9 PDZ
A9V6G5_MONBE	HRESTV	H**S**ET**A**L (TMIGD1)	97 ± 30
A9UWH1_MONBE	STRSDV	H**S**ET**A**L (TMIGD1)	>1000
A9UR52_MONBE	SRRTEV	**R**R**E**T**Q**V (HPV18 E6)	>1000
A9UWP5_MONVE	GSESSV	**A**S**G**SSV (SYNJ2BP)	490 ± 110
A9UYY4_MONBE	RLASEV	**VI**ATEV (DLL1/4)	>1000
A9VB85_MONBE	ARESEI		>1000
DLL1/DLL4	VIATEV		240 ± 40

**Table 5:** Example output of Motif Matcher, using sequence GGGTGL as a search model.

Number of Substitutions	UniProt ID	Matching Sequence	Reference Sequence
2	tr|A9UQN5|A9UQN5_MONBE	GGCTLL	GGGTGL
2	tr|A9V457|A9V457_MONBE	YGGTSF	GGGTGL
2	tr|A9UZY0|A9UZY0_MONBE	RYGSGV	GGGTGL
2	tr|A9UWI6|A9UWI6_MONBE	GGCSLL	GGGTGL
2	tr|A9UR91|A9UR91_MONBE	GYGSTI	GGGTGL
2	tr|A9UU72|A9UU72_MONBE	GGPTDI	GGGTGL
2	tr|A9UX10|A9UX10_MONBE	GLGTTI	GGGTGL
2	tr|A9UNL7|A9UNL7_MONBE	GGSTQI	GGGTGL
2	tr|A9UPF5|A9UPF5_MONBE	GDGSSF	GGGTGL

## Data Availability

All computational programs developed in this work are available at https://pdzselectivity.cs.wwu.edu (accessed on 11 September 2021).

## Sample Availability

Samples of the constructs used in this study are available by the authors.
